# Advanced Maternal Age Impairs Myelination in Offspring Rats

**DOI:** 10.3389/fped.2022.850213

**Published:** 2022-03-03

**Authors:** Wei Han, Ya'nan Pan, Ziyao Han, Li Cheng, Li Jiang

**Affiliations:** Department of Neurology Children's Hospital of Chongqing Medical University, National Clinical Research Center for Child Health and Disorders, Ministry of Education Key Laboratory of Child Development and Disorders, China International Science and Technology Cooperation Base of Child Development and Critical Disorders, Chongqing Key Laboratory of Pediatrics, Chongqing, China

**Keywords:** advanced maternal age, myelin sheath development, offspring, ERK, cognitive impairment

## Abstract

The effects of advanced maternal age (AMA) on the neurodevelopment of offspring are becoming increasingly important. Myelination is an important aspect of brain development; however, a limited number of studies have focused on the effects of AMA on myelination in offspring. The current study aims to evaluate the association between AMA and myelin sheath development in offspring. We studied the learning and memory function of immature offspring using the novel object recognition test. Then, we investigated the expression of myelin basic protein (MBP) in the immature offspring of young (3-month-old) and old (12-month-old) female rats at different time points (14, 28, and 60 days) after birth with immunofluorescence and western blotting. The myelin sheath ultrastructure was observed with transmission electron microscopy in immature and mature offspring. Extracellular signal-regulated kinase 1 and 2 (ERK1/2) and phosphorylated ERK1/2 (p-ERK1/2) were investigated by western blot in immature offspring at the above time points. AMA impaired the memory function of offspring during early postnatal days. The MBP expression level gradually increased with postnatal development in the offspring of both the AMA and Control (Ctl) groups, but the MBP level in the offspring of the AMA group was lower than that of the Ctl group at 14 days after birth. In addition, the ultrastructure of the myelin sheath was defective in AMA offspring during the early postnatal period; however, the myelin sheath was not significantly affected in offspring during adulthood. Interestingly, ERK phosphorylation at 14 days after birth was lower in AMA offspring than in Ctl offspring. However, ERK phosphorylation at 28 days after birth was higher in AMA offspring than in Ctl offspring. The peak of ERK phosphorylation in the AMA group was abnormal and delayed. Our results indicated that AMA is associated with poor developmental myelin formation in offspring. The ERK signaling pathway may play an essential role in the adverse effects of AMA on the offspring myelin sheath development.

## Introduction

According to the International Federation of Gynecology and Obstetrics (FIGO), women over 35 years old at the time of delivery are considered to have advanced maternal age (AMA). AMA has become increasingly prominent worldwide following socioeconomic development in the last decade. The impact of AMA on adverse maternal and offspring outcomes, such as spontaneous abortion, gene and chromosomal abnormalities, congenital malformations, preterm delivery, low birth weight, and stillbirth, has been extensively studied (1–4).

AMA is also associated with the neurodevelopment of offspring, which is of great importance and debate. Some epidemiologic studies have shown that AMA is correlated with multiple neurodevelopmental disorders, including cognitive disorders, autism, schizophrenia, bipolar disorder, dyslexia, and epilepsy (5–8). The effect of maternal age at conception on the cerebral performance of offspring has also been observed in AMA animal models (9), and our previous work showed that the offspring of mothers with AMA had worse cognitive function than offspring born to rats of ordinary reproductive age (10).

Early neurodevelopment is a highly dynamic process during which the structure and function of the brain evolve simultaneously. With the exception of synapse formation, myelination is perhaps the most important event that occurs during the early period of postnatal neurodevelopment. The myelin sheath, an important component of the central nervous system, is formed by mature oligodendrocytes that firmly attach to axons. The myelin sheath evolved to enable the rapid and efficient transmission of electrical signals in the nervous system, which is critical for interneural communication (11). Various conditions, such as hypoxia or autoimmune disorders, during development can impair central nervous system (CNS) myelination and lead to different damage characteristics during different developmental stages; thus, impaired myelination is an important cause of many white matter disorders in the brain (12–14). Myelination is recognized as one of the critical mechanisms underlying white matter plasticity. Furthermore, myelination has been linked to cognitive ability, and structural abnormalities in the myelin sheath could cause a variety of neurological conditions (15, 16). It is critical to study and clarify the pattern of myelination during development in the offspring of mothers with AMA to explain the mechanism of brain dysfunction in AMA offspring.

Because rats are often used as an animal model in research on CNS myelin diseases, we used a model consisting of the offspring of AMA rats, as our previous study (10). The rat brain is rapidly myelinated after birth; after approximately 2–3 weeks of postnatal (P) rapid myelination, the total amount of myelin reaches 50% of that of adult rats approximately 4 weeks after birth; 8 weeks after birth corresponds to the early adult stage in rats, when myelination is essentially complete; and P60 rats correspond to adults (17). Moreover, myelination is a complex and dynamic process that continues in humans well into adulthood, enabling the ability to regenerate myelin sheaths during adulthood (18, 19).

Myelin basic protein (MBP) is the most abundant in compact myelin and plays an important role in the assembly and suppression of myelin and influences the correct expression and distribution of other myelin proteins (19). Therefore, the expression and morphology of MBP can effectively reflect myelin sheath development and the degree of damage to the myelin sheath, which can be used to assess the effects on myelin.

During myelin development, myelin formation is regulated by signals from nerve axons. ERK has been described as a major signaling component of the mitogen-activated protein kinase (MAPK) signal transduction system that is widely involved in regulating cell survival, cell cycle entry, cell proliferation and cell differentiation. Several studies have shown that ERK plays an important role in regulating oligodendrocyte development, myelination formation and myelin sheath thickness (20–23).

In this work, we used an AMA rat model to investigate myelin sheath development in AMA offspring and report the underlying mechanism that may have implications for the prevention and treatment of brain disorders in offspring in the future.

## Materials and Methods

### Animals

Healthy male specific-pathogen-free (SPF) Sprague–Dawley (SD) rats were kept under a controlled environment condition (12 h:12 h light/dark cycle, temperature of 21 ± 1°C and humidity of 60%) with free access to food and water. We collected 12-month-old and 3-month-old female SD rats (*n* = 12 in each group) with no prior pregnancy experience. The virgin rats were mated individually with a randomly selected 3-month-old male rat. After pregnancy, female rats were removed from their cages for natural delivery. The offspring of 12-month-old rats were randomly selected from each litter and assigned to the AMA group (*n* = 45 in total). The offspring of 3-month-old female rats were randomly selected (both male and female) as the control group (Ctl; *n* = 45 in total) according to the quantity and sex of the AMA group (Supplementary Material Table 1). A novel object recognition behavior experiment was performed at P28 to assess cognitive function in the AMA offspring. At three time points (P14, P28, and P60), animals were sacrificed, and then the samples were prepared for immunofluorescence, western blot analysis and transmission electron microscopy. All animal experimental procedures were conducted in accordance with international guidelines for the care and use of laboratory animals and were approved by the Animal Care Committee of Chongqing Medical University, Chongqing, China.

### Novel Object Recognition Test

The Novel object recognition experiment was conducted at P28 to assess the cognitive function of the rats. The rats were placed in a 1 × 1 m square black box. Each experimental rat was placed in the tank for 10 min to allow it to adapt to the box environment to avoid interference during the test period. On day 1, two objects of the same size and color were placed in the two corners of the tank, and the rats were placed in the tank to become familiar with the two objects for 5 min. On day two, one object was replaced by another object whose shape and color were completely different from those of the object present on day 1. The rat was put into the box and allowed to move freely for 5 min. Effective contact was defined as directly touching the object or a distance of <2 cm from the object recorded by the nose of the rat. The time and numbers of rats touching the object were recorded by the computerized video tracking system. The discrimination ratio was calculated as the time spent exploring the new objects/time spent exploring the old objects.

### Immunofluorescence

At 14, 28, and 60 days after birth, rats (*n* = 5 rats for each group) were intraperitoneally (i.p.) anesthetized deeply with sodium pentobarbital and then perfused with 0.9% sodium chloride. Brains were removed and fixed in 4% paraformaldehyde. Each brain was divided into 30-μm-thick frozen coronal sections. Complete eyebrow-shaped hippocampal slices were taken from the same septotemporal level for MBP staining. Anti-MBP primary antibody (Cat. No:SMI-99P, mouse monoclonal antibody, Biolegend, San Diego, CA) was used at a concentration of 1:500. The primary antibody was incubated with the tissue overnight. Then, the slides were incubated with the Alexa Fluor 555 labeled donkey anti-mouse secondary antibodies (Cat. No: A0460, Beyotime biotechnology, Shanghai, China) at 1:500 for 2 h at room temperature away from light.A laser scanning confocal microscope was used to capture images with the same exposure time and blindly analyzed the average fluorescence intensity of MBP-positive cells in the hippocampi and surrounding callosum of rats using Nis-element BR3.2 software.

### Western Blot Analysis

The hippocampi of rats at different time points (P14, P28, and P60) were rapidly dissected and immediately stored in liquid nitrogen. The protein concentration was determined using a Bio–Rad protein assay kit (Bio–Rad Laboratories, USA). Samples containing 30 μg of total protein were loaded onto 12% sodium dodecyl sulfate (SDS)-polyacrylamide gels and electrophoretically separated. The proteins were then transferred to polyvinylidene difluoride membranes (0.22 μm, Millipore Corp, Billerica, MA, USA). The membranes were blocked for 1 h in 5% bovine serum albumin before incubation overnight at 4°C with the following specific primary antibodies: anti-MBP (Cat. No: SMI-99P, mouse monoclonal antibody, Biolegend, San Diego, CA, 1:1,000), anti-extracellular signal-regulated kinases 1 and 2 (Cat. No:4695, anti-ERK1/2, rabbit monoclonal antibody, Cell Signaling Technology, Massachusetts, USA, 1:2,000), anti-phospho-ERK1/2 (Cat. No:4370, anti-p-ERK1/2, rabbit monoclonal antibody, Cell Signaling Technology, Massachusetts, USA, 1:2,000) and anti-β-actin antibody (Cat. No:700068, mouse monoclonal antibody, horseradish peroxidase conjugate, Zen Bioscience, Chengdu, 1:1,000). The membranes were incubated with the corresponding secondary antibody (Cat. No: ZB-2305/ZB-2301, goat anti-mouse/rabbit IgG; ZSGB-BIO, Beijing, China, 1:5,000) for 90 min. Protein bands were visualized using clear western electrochemiluminescence (ECL) substrate (Bio–Rad). The protein bands were analyzed by densitometry using a Bio–Rad imaging system and Image Lab software (*n* = 6 in each group).

### Transmission Electron Microscopy

The rats (*n* = 3 in each group) were transcardially perfused with 0.9% sodium chloride and 2.5% glutaraldehyde in 0.2 M phosphate-buffered saline (PBS). Under an anatomical microscope, 1 mm^3^ tissue slabs were sampled from the CA1 region of the hippocampus, which was separated from the cerebrum, and fixed in 4% dedicated glutaraldehyde for 2 h at 4°C. Next, the samples were osmicated in osmium acid in 0.1 M PBS for 2 h at 4°C. The tissues were cut into single 60 nm sections using an ultramicrotome before being stained with uranyl acetate for 15 min and lead citrate for 2 min. A transmission electron microscope (Hitachi-7500P, Phillips, Ltd., Holland) was used to observe the ultrastructural morphological changes in hippocampal myelin. For each section, three fields of vision were randomly chosen and photographed at a magnification of 40,000 × .

The g-ratio, defined as the axon diameter (AD) to the nerve fiber diameter (FD), is a reliable index of myelination that is independent of axonal diameter (13, 14). We used g-ratios to evaluate the thickness of myelin sheaths. The diameters of the nerve fibers and axons were measured by hand tracing using ImageJ software, and then the areas from which the diameters were derived were calculated. Nine sections were randomly selected from each rat for analysis.

### Statistical Analysis

SPSS 17.0 software was used to analyze the data. All data are presented as the mean ± standard error of the mean (SEM). The results in the Ctl and AMA groups were compared and analyzed using an independent samples *t*-test at the same time point. For multiple comparisons, one-way analysis of variance (ANOVA) with Dunnett's T3 test was used. Statistical significance was considered if *p* < 0.05.

## Results

### AMA Impaired Learning and Memory Function in Offspring

**Figure 1:**
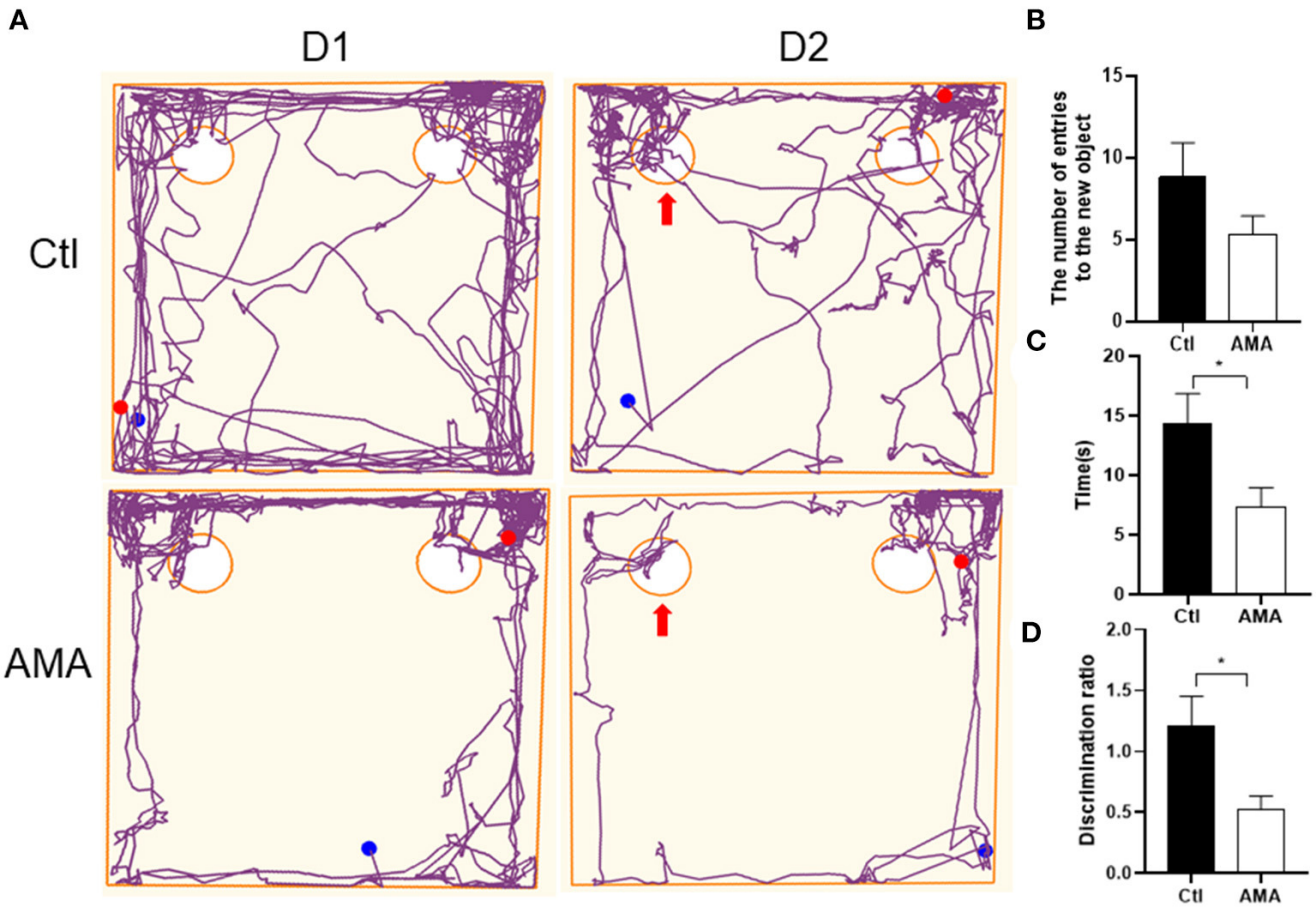
The discrimination ratio of the exploration time affected by AMA in the novel object recognition test. **(A)** Representative track maps in the novel object recognition test showed that offspring in Ctl spent more time around the novel object (as arrow shown) and reflected a better ability to explore new objects than offspring in the AMA group. **(B)** The number of the rat touched the novel object with its nose, **(C)** the time of the rat stood near the novel object, and **(D)** the discrimination ratio in the Ctl and AMA groups. All data are shown as the mean ± SEM, *n* = 6 in each group, *P < 0.05.

We first analyzed the offspring's learning and memory function by novel object recognition behavioral experiments (26). As shown in Figure 1A, compared with the Ctl group, offspring born to AMA rats had worse learning and memory function in the early postnatal period. We observed no significant difference in the number of rats touching the new object (Ctl vs. AMA: 8.33 ± 2.08 vs. 5.33 ± 1.11; *t* = 1.47, *P* = 0.17; Figure 1B). However, a significant difference was detected over time (Ctl vs. AMA: 14.43 ± 2.43 vs. 7.33 ± 1.62; *t* = 2.42, *P* = 0.036; Figure 1C) and discrimination ratio (Ctl vs. AMA: 1.21 ± 0.22 vs. 0.52 ± 0.10; *t* = 2.63, *P* = 0.025; Figure 1D) to explore new objects in Ctl and AMA offspring. Compared with the Ctl group, AMA offspring reflected a worse ability to explore new objects.

### The Effects of AMA on MBP Protein Expression in the Hippocampi of AMA Offspring

**Figure 2:**
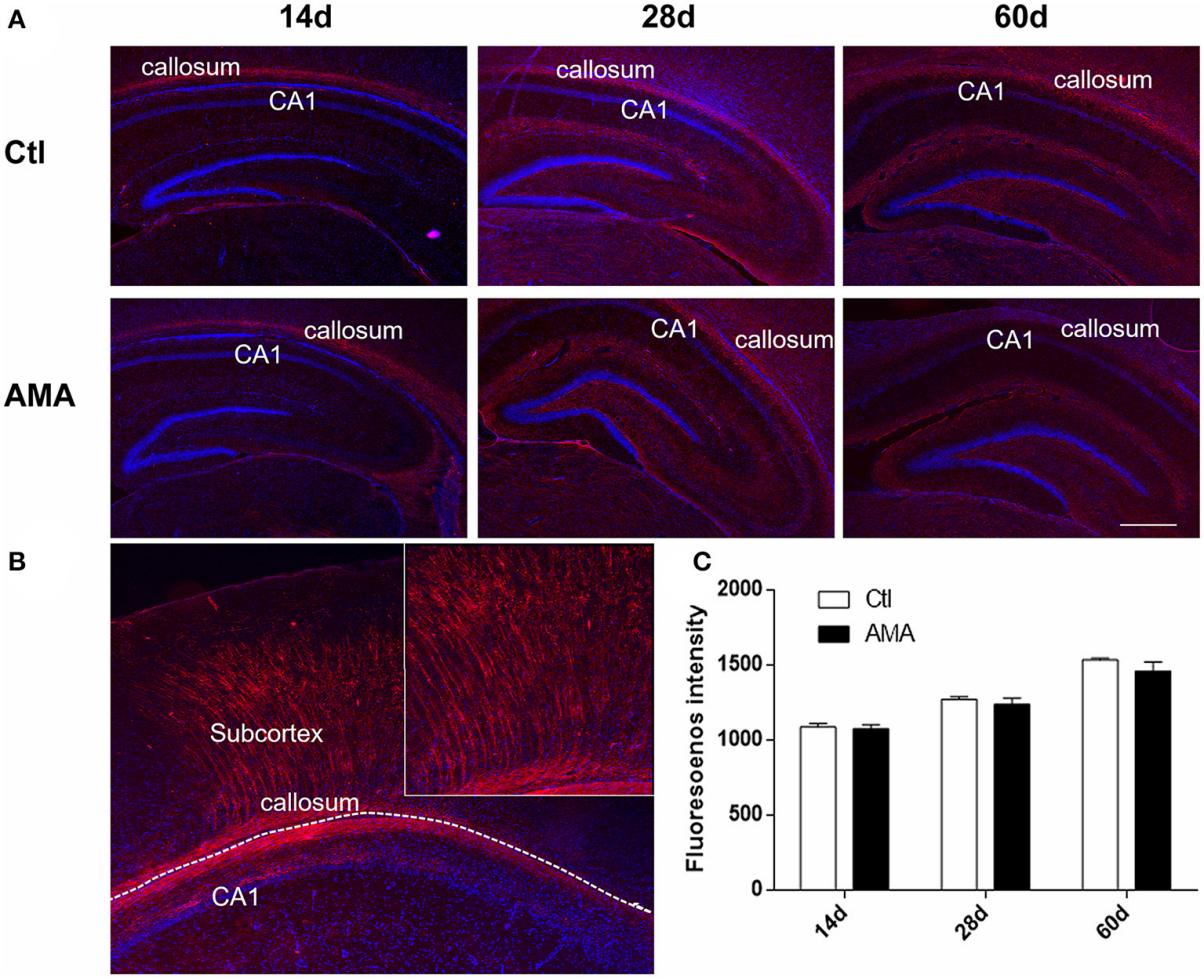
Immunofluorescence of MBP in the AMA and control groups. **(A)** Immunofluorescence showing the protein expression of MBP in hippocampal tissue samples collected from rats on P14, P28, and P60. **(B)** The shape of the MBP in the CA1, callosum and subcortex. **(C)** Quantitative analyses of the intensity of MBP-positive cells at different times for each group. The scale bar is 100 μm. Data are expressed as the mean ± SEM (*n* = 5 rats in each group).

MBP is one of the major proteins in compact myelin. To identify whether AMA can affect myelin sheath development in offspring, we labeled cells with MBP in the hippocampus with immunofluorescence, and the relative intensity was quantified to indirectly measure the myelin level. Normally, the myelin sheath matures gradually from childhood to adulthood, as shown in Figure 2A. Expression was initiated in the pleomorphic layer of the hippocampal CA1 region and corpus callosum on P14. Gradually, MBP appeared in the stratum lacunosum-moleculare and fimbria of the hippocampus on P28, and MBP was expressed in the corpus callosum, subcortex, and around the ventricle by P60. The expression site did not differ significantly between the AMA and Ctl groups.

Figure 2B, a representative graph on P28 of Ctl, indicates that MBP is typically expressed as fibrous strips in CA1 area and callosum which radiating to the subventricular white matter area. For the reason, the fluorescence intensity of MBP-positive in CA1 and surrounding callosum was calculated. As shown in Figure 2C, the intensity of MBP-positive cell colonies increased with age in the corpus callosum around the hippocampus, peaking on P60 in both the AMA (1,535.20 ± 10.57) and Ctl (1,458.41 ± 63.11) groups. The results showed that the fluorescence intensity of MBP was a lower trend in the AMA group than in the Ctl group at each time point, but the difference was not statistically significant (*t* = 1.2, *P* = 0.31).

**Figure 3:**
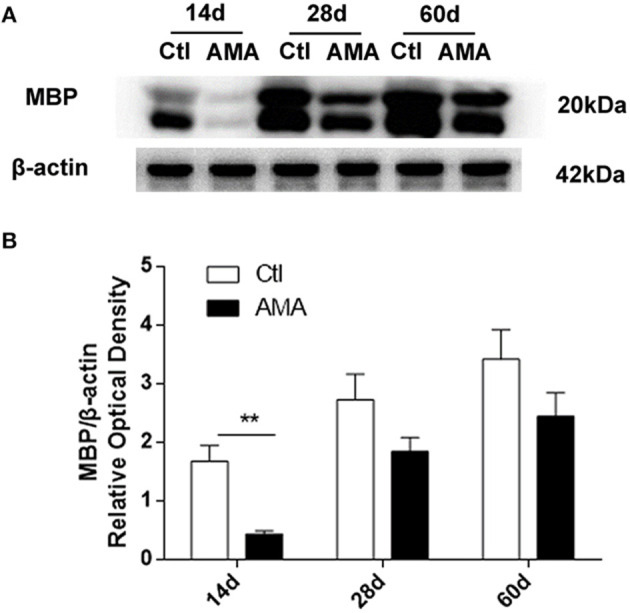
Effects of AMA on MBP protein expression levels in the hippocampus in different groups. **(A)** Representative protein bands of MBP in hippocampal tissue samples collected from rats on P14, P28, and P60. **(B)** Quantitative analyses of the MBP/β-actin expression level at different times for each group. Values are the mean ± SEM (*n* = 6 rats in each group), **p < 0.01.

To enhance the evidence of AMA on myelin sheath development deficits in offspring, we subsequently conducted western blotting to detect the MBP expression level. As shown in Figure 3A, the hippocampal expression of MBP in the AMA group was lower trend than that in the control group at all timepoints. In addition, as shown in Figure 3B, a significant difference in the MBP/actin expression ratio was observed in the hippocampus on P14 after birth relative to those in the Ctl group (Ctl vs. AMA: 1.68 ± 0.27 vs. 0.43 ± 0.55, *t* = 4.5, *p* = 0.005 on P14), indicating that myelin sheath damage was initiated in the immature brains of offspring rats during the early phase after birth and that the severity of this damage increased throughout the developmental period into adulthood.

### Ultrastructural Changes to the Myelin Sheath in the Hippocampi of AMA Offspring

**Figure 4:**
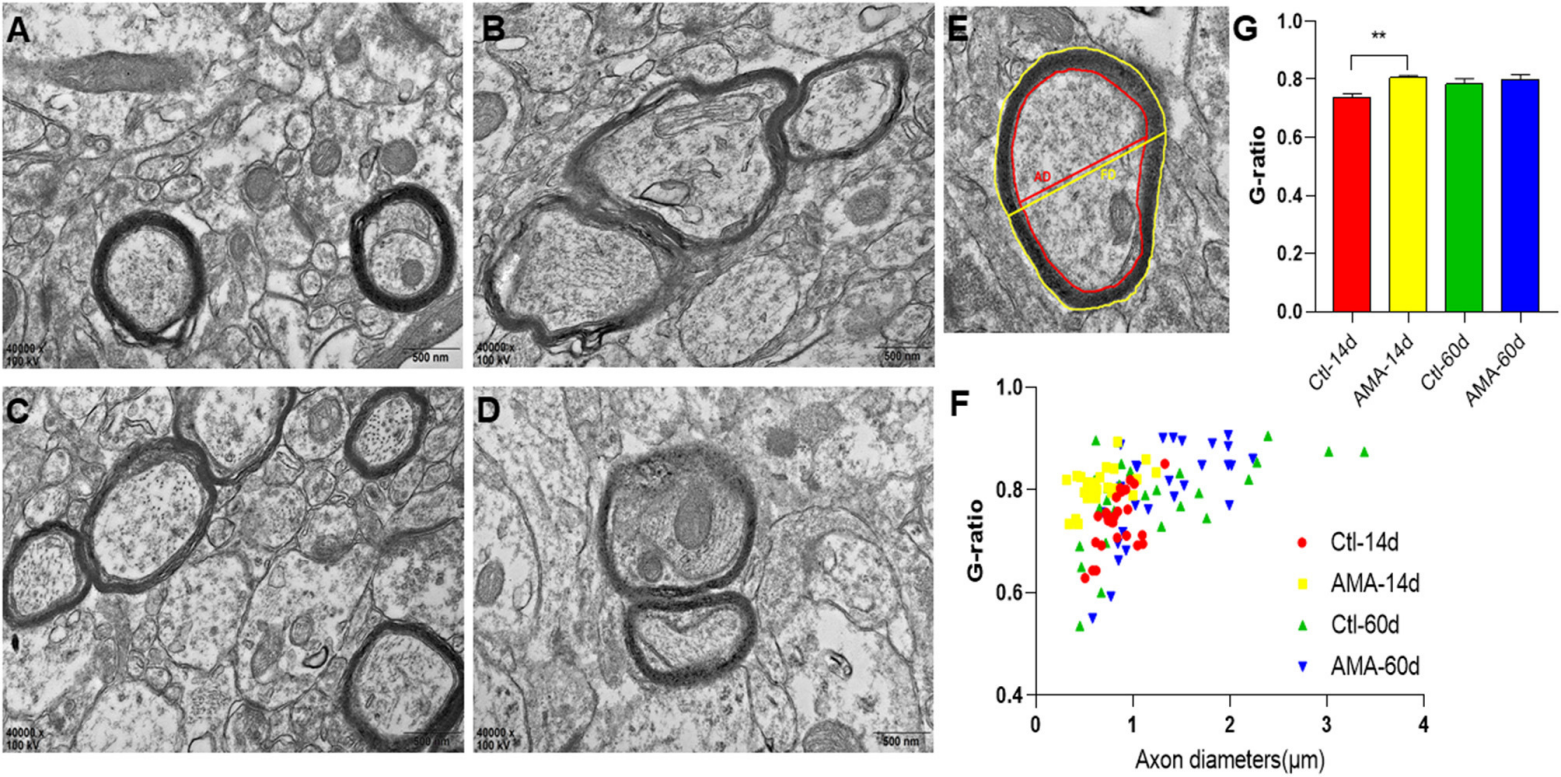
Ultrastructural Changes to Myelin Sheath in the AMA and control groups. **(A)** Representative transmission electron microscopy images of the ultrastructure of myelinated fibers in the hippocampi of rats in the Ctl and **(B)** AMA groups on P14. **(C)** Transmission electron microscopy revealed the ultrastructure of myelinated fibers in the hippocampi of rats in the Ctl and **(D)** AMA groups on P60 (×40,000, scale: 500 nm). **(E)** Examples of the measurements used for the analysis of the g-ratio. **(F,G)** Quantification of the myelinated g-ratio in the AMA and control groups. **p < 0.01, *n* = 3 rats in each group.

The hippocampal myelin sheath in rat offspring on P14 and P60 was observed using TEM. As previously mentioned, the critical time point for myelin formation in rats is 14 days after birth, and myelination is largely complete 60 days after birth. The myelin sheath in the Ctl groups contained neural fibers with regular thickness and a dense structure. However, the myelin in the AMA group showed varying degrees of stratification, vacuolation, collapse, disruption, and an irregular shape (Figures 4A–D) at days 14 and 60 after birth.

Myelin sheath thickness in different groups was measured by determining the g-ratios (Figures 4E–G). ANOVA revealed a significant difference in the g-ratio between the groups (*F* = 4.405, *P* = 0.006). The g-ratio in the Ctl group on P14 (0.74 ± 0.01) was significantly lower than that in the AMA group (0.81 ± 0.01). However, the g-ratio in the AMA group on P60 (0.79 ± 0.02) was not significantly different from that in the Ctl group (0.78 ± 0.02). This indicated that the myelin in the offspring of mothers with advanced maternal age was thinner during the early postnatal period after birth.

### The Effects of AMA on ERK Phosphorylation Levels in the Hippocampi of AMA Offspring

**Figure 5:**
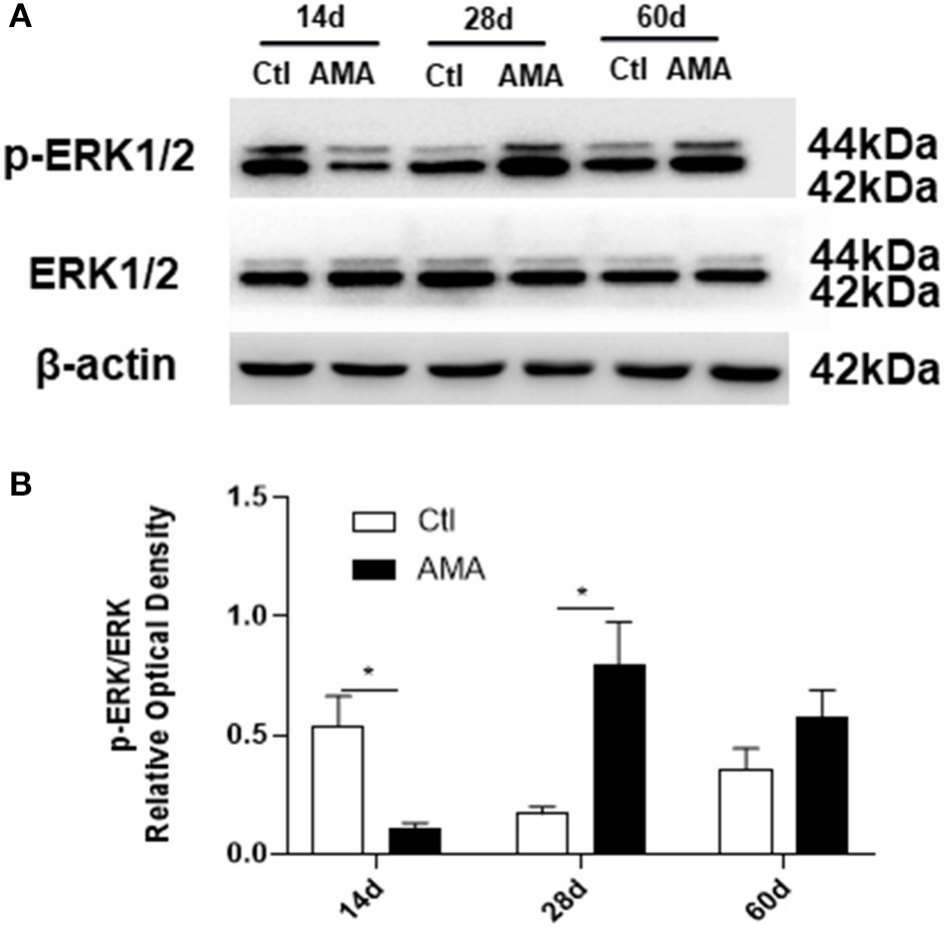
Effects of AMA on ERK1/2 phosphorylation in the hippocampi of AMA offspring. **(A)** Representative images showing the expression of ERK1/2 and p-ERK1/2 in hippocampal tissue samples collected from Ctl and AMA rats at three different time points (P14, P28, and P60). **(B)** Quantitative analyses of ERK phosphorylation at different times for each group. Data are shown as the mean ± SEM, *p < 0.05 compared with Ctl, *n* = 6 per group.

To determine whether the ERK pathway mediates AMA-induced injury to the myelin sheath in offspring, the protein expression levels of ERK1/2 and p-ERK1/2 in the hippocampus were measured using western blot analysis (Figure 5). The ratio of the p-ERK1/2/ERK1/2 protein expression intensities was used to represent the level of ERK phosphorylation.

In the control group, the p-ERK1/2 protein expression level dramatically peaked at 14 days, and then decreased with increasing age, reaching a relatively low level in adulthood. It is interesting to note that the p-ERK1/2 expression in the AMA group significantly lower than that in Ctl group at 14 days (Ctl vs. AMA: 0.54 ± 0.13 vs. 0.11 ± 0.02, *t* = 3.26, *P* = 0.021). The p-ERK1/2 expression at 28 days postnatal was sharply increased in the AMA group and was statistically different than the control group (Ctl vs. AMA: 0.17 ± 0.03 vs. 0.80 ± 0.18, *t* = −3.43, *P* = 0.017). However, the difference in p-ERK1/2 expression at 60 days was not significantly different between the AMA and control groups (Ctl vs. AMA: 0.36 ± 0.09 vs. 0.36 ± 0.09, *t* = −1.46, *P* = 0.16). These data indicate that AMA results in abnormal levels of ERK phosphorylation in the immature brains of rat offspring.

## Discussion

The overall prevalence of advanced maternal age (AMA) in a previous large multiple-country analysis was 12.3%, ranging from 2.8 to 31.1% (1). Offspring born to mothers with AMA have previously been shown to have a higher risk of brain development disorders, including cognitive disorders, depression, anxiety and emotional development disorders (24–26). The brain is one of the most important organs of the human body, and whether the brain develops normally during childhood has long-term consequences; thus, how to effectively prevent the occurrence of brain damage and mitigate or stop brain damage in offspring is a critical issue in the field of pediatric neurology.

Our results showed that AMA impaired immature offspring cognitive function, which is consistent with previous studies (10, 26). As for the brain development of the offspring, myelination is related to cognitive function. We observed that MBP expression in the hippocampus and corpus callosum increased with immature brain development and remained sufficient during adulthood. However, in the AMA group, MBP levels were lower than in the control group during early postnatal period. Moreover, AMA had an adverse effect not only on the structure and thickness of the myelin sheath in offspring during childhood but also on the myelination process. This observation indicated that AMA might affect the development and function of the myelin sheath during the early stages of development in offspring and that this negative impact continues into adulthood. Based on our data, we speculate that the myelin sheath is critical in the pathogenesis of the effect of AMA on white matter abnormalities and neurocognitive development in offspring.

Increasing evidence indicates that myelination of the first axons does not start until 30 weeks gestational age and that most myelination occurs postnatally during the first year of life (14, 27, 28). Infants are vulnerable to white matter injury because crucial processes in white matter development occur during late pregnancy and the early postnatal period. It has been suggested that myelination is affected at different stages of development and manifests differently, leading to the development of cerebral white matter disease (29). Interestingly, our data indicated that the most severe influence on myelin development in AMA offspring was during the early postnatal period of the immature brain and that delayed myelination might lead to neurological problems later in life, providing us with a potential window of time for intervention due to the plasticity of the developing brain.

ERK1/2 signaling has been proposed to play a dominant role in the control of survival, proliferation and differentiation in oligodendrocyte precursor cells (OPCs), a type of glial cell thought to be the primary source of oligodendrocytes (23). Importantly, Erk1/2 signaling exerts a highly temporally specificity that only at the earliest stages of OPC development could promote OPC proliferation (30). Moreover, studies have shown that ERK1/2 signaling exerts a direct effect on myelination independent of regulating OPC during brain development, which continues throughout adulthood. Conditional activation of Erk1/2 would be beneficial for enhancing new myelin wraps, functional changes and dramatically increases myelin sheath thickness during developmental myelination (31, 32). Evidence has demonstrated that the temporally correct moment of promyelinating signals is crucial for successfully myelinate due to the time window to initiate the process of myelination is relatively limited (33). Based on our data, the peak ERK1/2 signaling activation in AMA offspring during postnatal development was delayed, which suggests that abnormal activation or disruption of the ERK pathway might the potential mechanisms for the delayed and abnormal myelination during development in AMA offspring. Nevertheless, more careful investigation is needed to determine how ERK impacts myelination in AMA offspring and how ERK promotes OPCs to oligodendrocytes differentiation during brain development in AMA offspring. Furthermore, several studies have identified Erk1/2 signaling cross talk with other pathways in promoting myelination including the serine/threonine kinase AKT, the serine/threonine kinase Fyn and the glycogen synthase kinase-3 (GSK3) (34–36). Whether other signaling cross talk with ERK involved in AMA myelination is a question for further study.

Myelination is quite a complex process, many signaling molecules have been implicated in regulating OPC proliferation, migration, and differentiation, such as the platelet-derived growth factor (PDGF) pathway, Notch pathway, Wnt/β-catenin pathway, BMP4 pathway, target of rapamycin signaling (mTOR) pathway, phosphoinositide 3-kinase (PI3K) pathways and thyroid hormone pathway, in addition to the ERK pathway (37–41). It is still unclear how childbearing age affects myelin sheath development in offspring through molecular mechanisms, but previous studies have indicated that relevant signaling pathways in OPC development are important to investigate further to reveal the pathogenesis of AMA and to provide clinical treatment plans.

In conclusion, we investigated the effects of AMA on cognitive function and myelin sheath development in offspring rats and found that ERK signaling might influence myelination during myelin sheath development in AMA offspring. Our results might provide experimental data for the prevention of AMA-related brain development and functional disorders in offspring in the future. Despite the novelty of these preliminary findings, our study has some limitations. It is worth noting that we have not thoroughly investigated the underlying mechanisms, which may be a future research direction. More research is needed to identify appropriate and timely interventions to reduce the impact of AMA on pregnancy outcomes.

## Data Availability Statement

The raw data supporting the conclusions of this article will be made available by the authors, without undue reservation.

## Ethics Statement

The animal study was reviewed and approved by the Ethics Committee of Children's Hospital of Chongqing Medical University (CHCMU-IACUC20210625001).

## Author Contributions

WH and LJ conceived the experiments. WH, YP, and ZH conducted the experiments and analyzed the raw data. LC provided reagents and materials. WH contributed to the original draft preparation. All authors have reviewed the results and agreed to the published version of the manuscript. All authors contributed to the article and approved the submitted version.

## Funding

This work was supported by grants from the National Natural Science Foundation of China (81873792), the Postdoctoral Science Fund Project of Chongqing Natural Science Foundation (cstc2021jcyj-bshX0243), the Youth Basic Research Project from Ministry of Education Key Laboratory of Child Development and Disorders (YBRP-202110), and the Science and Technology Research Program of Chongqing Municipal Education Commission (KJQN202100423). The study was also supported by special funding of Chongqing postdoctoral research projects for WH.

## Conflict of Interest

The authors declare that the research was conducted in the absence of any commercial or financial relationships that could be construed as a potential conflict of interest.

## Publisher's Note

All claims expressed in this article are solely those of the authors and do not necessarily represent those of their affiliated organizations, or those of the publisher, the editors and the reviewers. Any product that may be evaluated in this article, or claim that may be made by its manufacturer, is not guaranteed or endorsed by the publisher.
