# Supplementary material for: Advanced Maternal Age Impairs Myelination in Offspring Rats
Source: Front Pediatr. 2022 Mar 3;10:850213. doi: 10.3389/fped.2022.850213 (PMC8927774; doi:10.3389/fped.2022.850213)
Supplement: Supplementary file 1 [file Data_Sheet_1.docx]

Supplementary Material

Table. 1 The fertility ability between the AMA and control groups

| Groups | N | Number of infertile rats | Litter size | Percentage of males (%) | male enrolled in present study | female enrolled in present study |
| --- | --- | --- | --- | --- | --- | --- |
| Ctl | 12 | 0 | 8.08±3.05 | 50.15±13.29 | 23 | 22 |
| AMA | 12 | 2 | 7.20±2.14 | 53.52±14.09 | 23 | 22 |

Note: Values are expressed as the mean ± SD.
